# The Clinical Contribution of Full-Field Electroretinography and 8-Year Experiences of Application in a Tertiary Medical Center

**DOI:** 10.3390/jpm11101022

**Published:** 2021-10-12

**Authors:** Jung-Je Yang, Chu-Hsuan Huang, Chang-Hao Yang, Chung-May Yang, Chao-Wen Lin, Tzyy-Chang Ho, Chang-Ping Lin, Yi-Ting Hsieh, Po-Ting Yeh, Tso-Ting Lai, Pei-Lung Chen, Ta-Ching Chen

**Affiliations:** 1 Department of Medical Education, National Taiwan University Hospital, Taipei 10002, Taiwan; b02401017@g.ntu.edu.tw; 2 Department of Ophthalmology, Cathay General Hospital, Taipei 10002, Taiwan; charleswain.h@gmail.com; 3 Department of Ophthalmology, National Taiwan University Hospital, Taipei 10002, Taiwan; chyangoph@ntu.edu.tw (C.-H.Y.); chungmay@ntu.edu.tw (C.-M.Y.); b91401108@ntu.edu.tw (C.-W.L.); hotchang@ntu.edu.tw (T.-C.H.); cpiling59@gmail.com (C.-P.L.); ythyth@gmail.com (Y.-T.H.); ptyeh67@ntu.edu.tw (P.-T.Y.); b91401005@ntu.edu.tw (T.-T.L.); 4 Department of Ophthalmology, College of Medicine, National Taiwan University, Taipei 10002, Taiwan; 5 Graduate Institute of Clinical Medicine, College of Medicine, National Taiwan University, Taipei 10002, Taiwan; paylong@gmail.com; 6 Graduate Institute of Medical Genomics and Proteomics, College of Medicine, National Taiwan University, Taipei 10002, Taiwan; 7 Department of Medical Genetics, National Taiwan University Hospital, Taipei 10002, Taiwan

**Keywords:** electroretinography, retinal dystrophies, retinal and macular diseases, optic neuropathies, systemic diseases

## Abstract

Electroretinography (ERG) is an important and well-established examination for retinal and visual pathway diseases. This study reviewed the medical records of patients who received full-field ERG (ffERG) at a single medical center between 2012 and 2019, which was an 8-year experience in the clinical contribution of ERG. Based on the indication for scheduling ffERG and the final diagnosis, patients could be classified into six groups: ‘retinal dystrophies’, ‘other retinal or macular diseases’, ‘optic neuropathies’, ‘visual complaints’, ‘systemic diseases’, and ‘others’. A total of 1921 full-field electroretinograms (ffERGs) (1655 patients) were included. The average number of ffERGs performed per year was 262 and the number of annual ffERGs was constant. The ‘retinal dystrophies’ group accounted for 36.5% of the studied population, followed by the ‘other retinal or macular diseases’ group (20.2%). The most common systemic disease was central nervous system disease. The rates of abnormal ffERGs in the ‘systemic diseases’, ‘optic neuropathies’, and ‘visual complaints’ groups were 27.3%, 22.6%, and 10.1%, respectively (*p* < 0.001). Higher rates were found in patients <20 years old in the ‘systemic diseases’ and ‘optic neuropathies’ groups; epilepsy and optic nerve atrophy were the most common diagnoses, respectively. In brief, by quantifying the functional response in the retina, ffERG is indispensable for diagnosis and prognosis in ophthalmologic and multidisciplinary practice.

## 1. Introduction

The application of electrophysiological examinations is long-standing and essential to diagnosing diseases of the retina and visual pathway. Full-field electroretinography (ffERG) is a well-established, non-invasive technique to assess comprehensive retinal function subjectively, using corneal electrodes to record electrical activity in photoreceptors and glial cells responding to light stimuli. Hence, ffERG is valuable in evaluating the physiology and integrity of the retina by its nature as a functional test and is useful for the diagnosis of wide-ranging retinal disorders [1,2].

Diagnosing specific retinal diseases, such as inherited retinal dystrophies, is challenging in the early stages due to non-pathognomonic clinical presentations and minimal changes in imaging examination. Electroretinography (ERG) plays an important role in demonstrating retinal dysfunction in the visual system to achieve an early diagnosis [3]. Based on the summation of electrical activity in the whole retina under a specifically set stimulus, characteristic changes in the ffERG can be detected and aid in differentiating retinal diseases affecting different retinal cells [4,5]. Different ERG subtypes other than ffERG have been employed in clinical conditions, including multifocal ERG and pattern ERG [2,6,7], which provide detailed information with higher sensitivity and specificity in particular retinal diseases. Particularly in the setting of inherited diseases, genetic counseling can be provided at an earlier stage due to the early identification of characteristic functional deficits, thereby directing the appropriate intervention to rescue vision or provide rehabilitation to maintain function in daily life.

Imaging technologies have also advanced significantly during the past decade. The development of optical coherent tomography (OCT) provides the ability to reveal detailed retinal structures and has changed the process of making a diagnosis and treatment plan [8]. Owing to the progress in novel methodologies, including spectral-domain OCT, swept-source OCT, and OCT angiography [8,9,10], a higher resolution providing more anatomical information is available within a shorter examination time. While ophthalmologists rely on modern imaging techniques to diagnose and confirm structural abnormalities, we are interested in understanding how ffERG, as an examination for functional response in the whole retina, complements multimodal retinal imaging in modern clinical applications.

In the present study, we aimed to investigate the real-world applications and diagnostic value of ffERG during the past eight years at the National Taiwan University Hospital (NTUH), which is one of the most well-established tertiary referral medical centers in Taiwan.

## 2. Materials and Methods

This was an observational, retrospective study that reviewed consecutive patients who underwent ffERG examinations between September 2012 and December 2019 at NTUH. The study was approved by the Institutional Review Board of the NTUH and adhered to the Declaration of Helsinki. Age, sex, clinical presentations, indication for arranging ffERG, and final diagnosis were recorded. The final diagnoses were based on clinical details and comprehensive ophthalmological examinations and were reviewed by a single ophthalmologist (TC Chen). We classified all patients into six groups based on the indication for arranging ffERG and the final diagnoses, which were ‘retinal dystrophies’, ‘other retinal or macular diseases’, ‘optic neuropathies’, ‘visual complaints’, ‘systemic diseases’, and ‘others’. Patients with visual complaints but without a definite diagnosis were included in the ‘visual complaints’ group. Patients who were recruited by other clinical studies, who had visual disturbance suspected to be associated with trauma or medication, or who were under evaluation for corneal transplantation, were classified as ‘others’.

### 2.1. ffERG Recording

All ffERGs were recorded according to the standard protocols of the International Society for Clinical Electrophysiology of Vision (ISCEV) of 2008 update and 2015 update [11,12]. Each patient received topical 0.5% proparacaine for anesthesia, and the pupils were dilated using topical 1% tropicamide. ERG recordings were obtained with ERG-Jet corneal contact lens electrodes (Fabrinal SA, La Chaux-de-Fonds, Switzerland) and reference electrodes over the scalp. The stimulation was provided by a Sunburst Ganzfeld stimulator (Utas Sunburst; LKC Technologies, Gaithersburg, MD, USA) with an Utas Visual Electrodiagnostic system. Dark-adapted series were made after 20 min of dark adaptation with a dim flash of 0.01 candela second (cd·s)/m^2^ (dark-adapted 0.01) and a bright flash of 3.0 cd·s/m^2^ (dark-adapted 3.0). After 10 min of light adaptation, a bright flash stimulus of 3.0 cd·s/m^2^ with a background luminance of 30 cd/m^2^ was applied as a single flash (light-adapted 3.0) and a 30 Hz flicker (light-adapted 3.0 30 Hz). No averaging was performed.

### 2.2. ffERG Data Interpretation

Patient data were compared with the department’s normative data from the normal population using the same testing equipment and protocols at the electrophysiological laboratory of the Department of Ophthalmology, NTUH [13]. Normative ffERG ranges were obtained separately for different age groups, which were stratified as 0–20, 20–40, 40–60, and >60 years of age. By modifying the threshold settings of previous studies, an abnormal ffERG response was defined as a reduction of more than 20% of the age-adjusted normative limit in amplitude, or a delay of more than 20% of the age-adjusted normative limit in implicit time [13,14,15,16]. Accordingly, a response would be defined as abnormal if an abnormal response was found for at least one of the three ffERG components, including cone function (assessed with light-adapted 3.0 and 3.0 30 Hz), rod function (assessed with dark-adapted 0.01 and 3.0), and the amplitude summation of oscillatory potential. Statistical analyses were performed using Excel (16.0.4266.1001; Microsoft Corporation, Albuquerque, NM, USA).

## 3. Results

**Figure 1:**
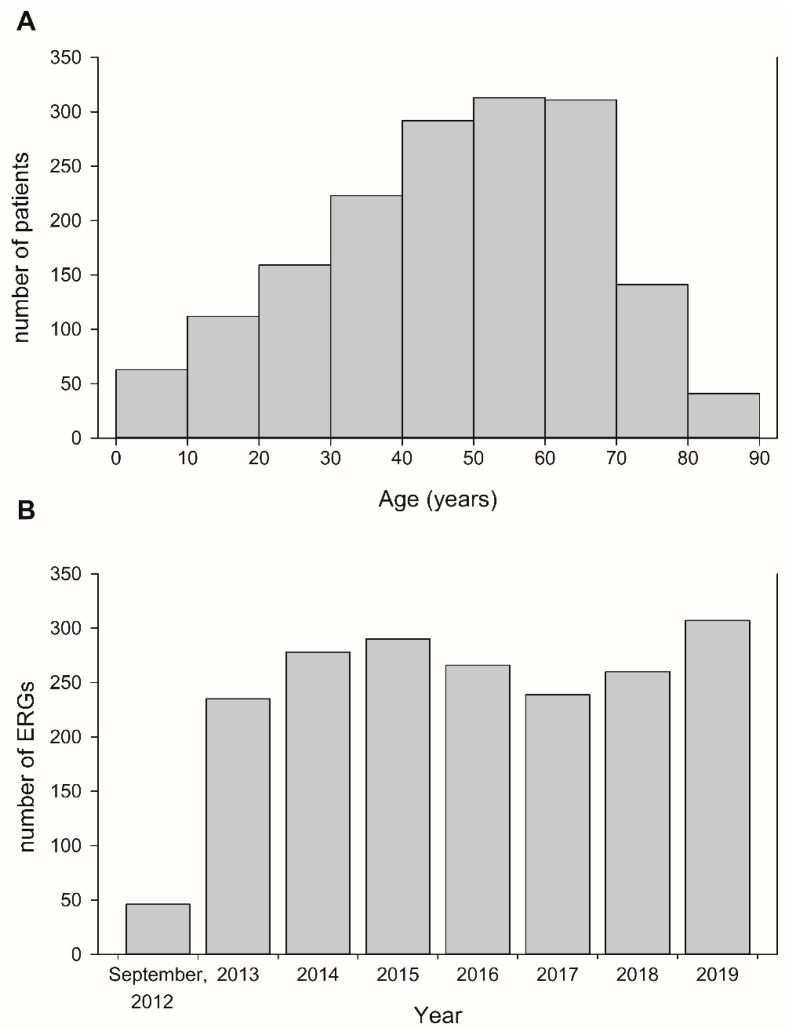
(**A**) Distribution of patients’ ages when receiving their first ffERG during the study period. (**B**) Cumulative number of ffERG examinations per year in this single tertiary medical center.

A total of 1921 ffERGs from 1655 patients (730 men and 925 women) were included in this retrospective study. The mean age of the patients was 47.4 years (range, 0.4–90.3 years) when the first ffERG was recorded. The age distribution of the patients is shown in Figure 1A. ffERGs were performed most frequently in patients aged between 50 and 60 years (18.9%, 313/1655), and 55.3% (916/1655) of ffERGs were performed in patients aged between 40 and 70 years. From September 2012 to November 2019, the average number of ffERGs performed was 262 times per year, and the number of annual ffERGs was constant during the study period (Figure 1B).

**Figure 2:**
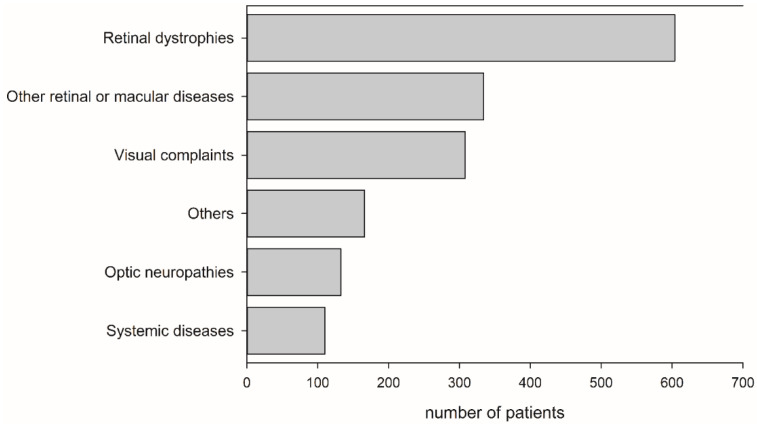
Cumulative numbers of patients in six diagnosis groups from September 2012 to December 2019 in his single tertiary medical center.

The cumulative number of patients in the six diagnosis groups are shown in Figure 2. Among the six groups, retinal dystrophies were the most prevalent diagnoses, accounting for 36.5% of patients (604/1655). The detailed diagnoses associated with these six groups and their age distributions when they received ffERG are listed in Table 1.

In the ‘retinal dystrophies’ group, retinitis pigmentosa (RP) was the most common diagnosis, accounting for 60.8% (367/604), followed by macular dystrophy (14.1%, 85/604) and cone–rod dystrophy (5.0%, 30/604). There were 35 patients who received ffERG in their first decade in this group, and 18 of them (51.4%) were diagnosed with Leber’s congenital amaurosis.

In the ‘other retinal or macular diseases’ group, age-related macular degeneration (AMD) (24.6%, 82/334) was the leading diagnosis, followed by vitreomacular disorders (21.0%, 70/334). In some retinal diseases, such as AMD, though it is not mandatory to diagnose these diseases with ffERG, ffERG was sometimes valuable in differentiating them from some other diffuse photoreceptor diseases, such as cone–rod dystrophies when there is bilateral and nearly symmetric involvement. ffERG may be also applied in cases with visual loss, which is disproportionate to macular findings, and some may have been due to undetermined visual impairment. There were 57 patients (17.1%, 57/334) in the cancer-related subgroup who were referred from oncological or hematological clinics because of unexplainable deteriorated vision. Lung cancer was the most common underlying malignancy (43.4%, 23/57). Among them, 25 patients were eventually confirmed with a diagnosis of cancer-associated retinopathy (CAR), and 30 patients had relatively normal ffERGs and lacked a definite diagnosis of CAR. The other two patients had infiltrated retinopathy with hematological malignancy.

There were another 133 patients with disorders involving optic nerve assorting in the ‘optic neuropathies’ group, including glaucoma, optic nerve atrophy, ischemic optic neuropathy, compressive optic neuropathy, and radiation-related optic neuropathy. ffERG was performed in these patients mostly to rule out retinal dysfunction.

ffERGs were often arranged for patients who reported visual disturbance but failed to show diagnostic clues in other imaging studies; these were included in our ‘visual complaints’ group if pathognomonic features were not detected. Most patients had non-specific visual disturbances (80.8%, 249/308), followed by night blindness (8.8%, 27/308). In addition, patients with amblyopia with undetermined etiology and who were suspected of having malinger tendencies were also tested by ffERG (7.1%, 22/308, and 3.2%, 10/308, respectively).

In the ‘systemic disease’ group, the majority of patients received ffERG because of neurological disease (89.1%, 98/110) involving the central nervous system, including cerebrovascular disease, brain tumors, epilepsy, Parkinson’s disease, and Wilson disease. Among the adolescent patients under 18 years of age in this group, 63.6% (7/11) were identified as having epilepsy. By defining the visual potential and retinal function, we cooperated with our neurological department to provide comprehensive medical care. In addition, ffERGs were also arranged for patients with autoimmune diseases, endocrine diseases, mitochondrial diseases, metabolic diseases, and other rare diseases.

In the ‘others’ group, all ffERGs were arranged by physicians other than retinal specialists in our hospital. Participants in clinical studies that required ffERG for assessment of retinal function constituted 47.6% (79/166) of cases in this group and were mostly recruited in clinical trials studying newly developed pharmacological products or cancer-related trials. ffERGs were performed in patients receiving specific medications for monitoring drug toxicity (40.4%, 67/166), mostly hydroxychloroquine (73.1%, 49/67) and psychotropic drugs (22.4%, 15/67). Patients who suffered from ocular or head trauma also underwent ffERGs to determine whether retinal function was affected (8.4%, 14/166). ffERGs were ordered by corneal ophthalmologists to evaluate the patients’ vision prognoses before corneal transplantation (3.6%, 6/166).

**Figure 3:**
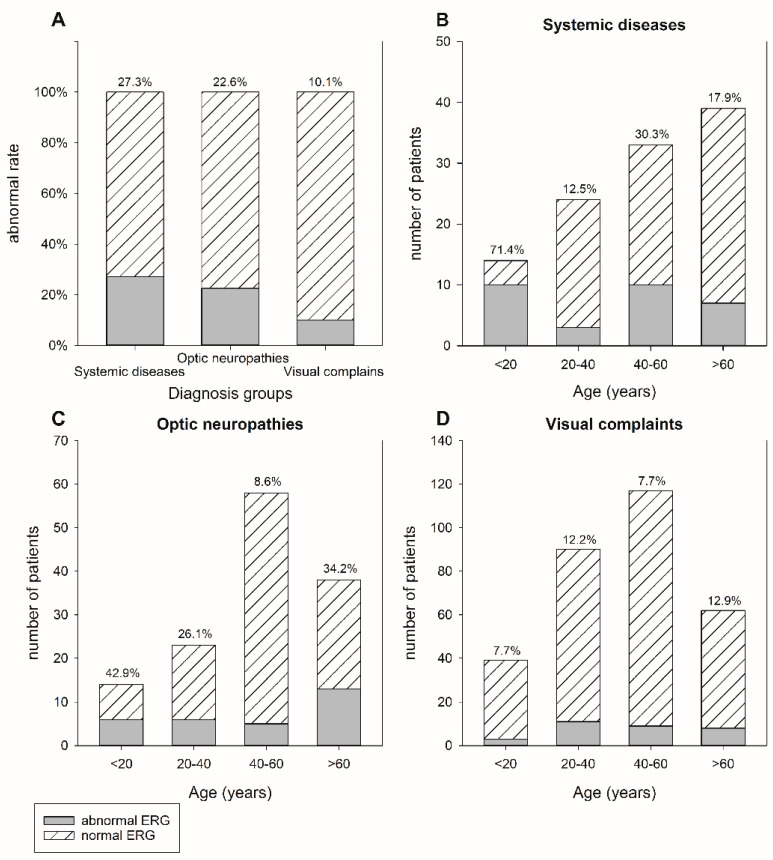
(**A**) Rates of abnormal ffERG examination results in the ‘systemic diseases’, ‘optic neuropathies’, and ‘visual complaints’ groups. (**B**–**D**) Rates of abnormal ffERG examination results and the age distribution of patients in the ‘systemic diseases’, ‘optic neuropathies’, and ‘visual complaints’ groups, respectively, when receiving their first ffERG.

The use of ffERG in the clinical diagnosis of retinal dystrophies and other maculopathies and retinopathies is well established. Hence, we further investigated the rates of abnormal ffERGs in the ‘systemic diseases’, ‘optic neuropathies’, and ‘visual complaints’ groups in order to reveal the role of ffERG. We found that 27.3% (30/110), 22.6% (30/133), and 10.1% (31/308) of patients presented with abnormal ffERGs in these three groups, respectively (*p* < 0.001 by the Chi-squared test, Figure 3A). The distribution of abnormal rates after stratification by age in different groups is shown in Figure 3B–D. Of the patients younger than 20 years, 71.4% (10/14) exhibited abnormal ffERGs in the ‘systemic disease’ group. Among them, five patients were confirmed to have developmental delay with underlying epilepsy or cerebral palsy. Syndromic diseases were highly suspected in the other five patients. In the ‘optic neuropathies’ group, most abnormal ffERGs were found in patients older than 60 years (34.2%, 13/38). Among them, eight patients (61.5%, 8/13) had glaucoma, three had optic nerve atrophy (23.1%, 3/13), and the other two patients had ischemic optic neuropathy and compressive optic neuropathy, respectively. In contrast, the subgroup of patients under 20 years of age exhibited the highest abnormal rate (42.9%, 6/14). The diagnoses were optic nerve atrophy in five patients and optic neuritis in one patient.

## 4. Discussion

This retrospective study describes our 8-year experience in performing ffERGs for variable clinical indications at a tertiary referral medical center. With rapidly developing optical imaging technologies, the precise retinal structure is accessible and revolutionizes the assessment of patients with retinal disorders [8,9]. Regardless, the diagnostic value from physiological assessment of the retina renders ffERG indispensable for diagnosing retinal dysfunction [17,18,19,20,21]. In the present study, we demonstrated the consistency and importance of ffERG in ophthalmological practice (Figure 1B).

### 4.1. Evaluating Retinal Function and Confirming the Diagnosis

**Figure 4:**
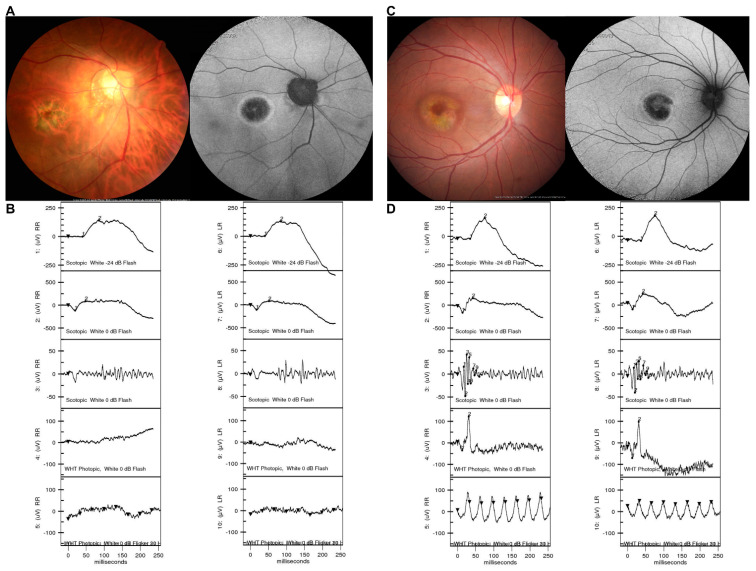
Representative cases illustrating the diagnostic value of ffERG in differentiating cone dystrophy and macular dystrophy. (**A**) Color fundus image and fundus autofluorescence in a 42-year-old male patient eventually diagnosed with cone dystrophy, presenting with central hypo-autofluorescence surrounded by a hyper-autofluorescent ring, resembling bull’s eye maculopathy. (**B**) ffERG in the same patient demonstrated abnormal light-adapted 3.0 and light-adapted 3.0 30 Hz series, which confirmed the diagnosis of cone dystrophy. (**C**) Color fundus image and fundus autofluorescence in a 21-year-old female patient eventually diagnosed with macular dystrophy, presenting with similar patterns as bull’s eye maculopathy. These images were not helpful for differentiation from the previous patient. (**D**) ffERG in this patient demonstrated normal cone and rod responses, indicating a diagnosis of macular dystrophy, which could be clearly differentiated from cone dystrophy using ffERG.

In the ‘retinal dystrophies’ group and the ‘other retinal or macular diseases’ group, most of the patients had provisional diagnoses after acquiring image examinations including fundus photography, fluorescein angiography, and OCT. ffERGs are essential for evaluating the extent of retinal dysfunction by quantifying the amplitude and implicit time. Characteristic ffERG patterns along with the imaging examinations confirmed the diagnoses (Figure 4). Moreover, ffERGs were helpful in subsequent visits as a reference for tracking disease progression as well [22,23]. In contrast, provisional diagnoses are more difficult to establish when the patients are too young to cooperate with physicians, or when in the early stage of disease with minimal changes in anatomical structure, which are virtually undetectable in ordinary studies [3,17]. ffERG plays an important role in collecting information and enhancing the diagnosis in these situations. For example, ffERG in severely visually impaired children is able to demonstrate typical abnormalities that support the diagnosis of Leber’s congenital amaurosis, which accounts for a large portion of blindness during the first decade of life. Therefore, the timing of clinical diagnosis facilitates molecular diagnosis and early rehabilitation, and might possibly permit genetic therapy to rescue visual function [3,24]. In some cases of retinal or macular diseases, ffERGs were used to confirm the baseline retinal function and to rule out the possible differential diagnoses. Subnormal or abnormal ffERGs may indicate a poor visual prognosis [20,25]. Moreover, multifocal ERG could further assist in the evaluation for maculopathy; for example, in patients with long-term use of specific medications, such as hydroxychloroquine, multifocal ERG is the standard tool for diagnosing and monitoring drug-associated retinal toxicity.

### 4.2. Identifying Further Diagnostic Clues

**Figure 5:**
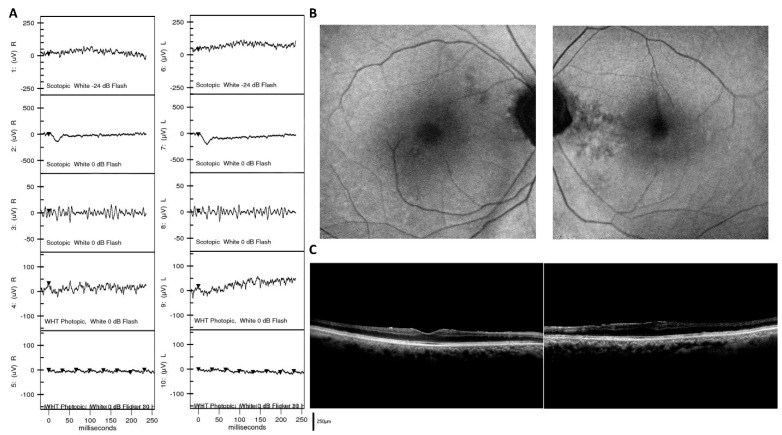
Multimodal images in a 79-year-old male patient with underlying hepatocellular carcinoma (HCC) presented with unexplanatory progressive vision loss and dim vision. (**A**) ffERG showed a severely attenuated response in both cones and rods. (**B**) Fundus autofluorescence did not reveal obvious retinal lesion or pathology. (**C**) OCT revealed generalized retinal thinning with central macular thickness 241 μm in his right eye and 268 μm in his left eye. HCC has a high prevalence in Taiwan, and this case demonstrated the possibility of HCC-related cancer-associated retinopathy (CAR).

**Figure 6:**
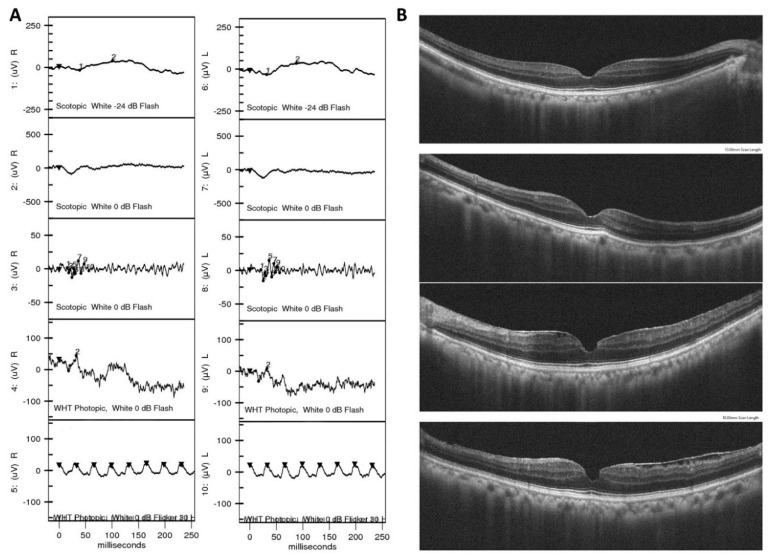
OCT and ffERG of a 72-year-old male patient who was molecularly diagnosed with CADASIL. Typical pathological findings in the central nervous system including ischemic stroke, intracerebral hemorrhage, and vascular dementia were noted, and we further found decreased amplitude in almost every mode of ffERG (**A**), which demonstrated degenerative changes in retinal neurons. OCT exam revealed relatively well-preserved architecture, except for a minor epiretinal membrane in the left eye (**B**).

**Figure 7:**
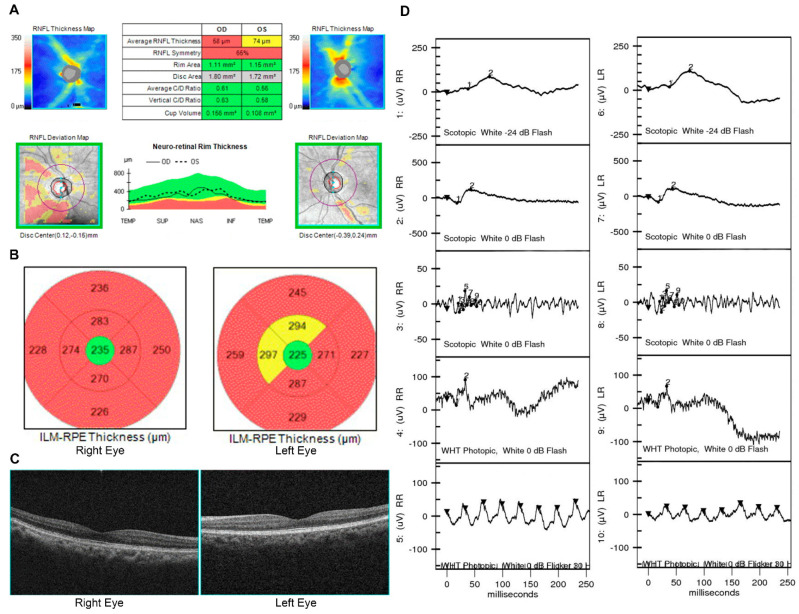
Multimodal images of a 67-year-old female patient diagnosed with advanced glaucoma and optic nerve atrophy. (**A**) OCT revealed enlarged optic cupping and a severely decreased retinal nerve fiber layer (58 μm in right eye and 74 μm in left eye), corresponding to the diagnosis of glaucoma and optic atrophy. (**B**,**C**) Moreover, a decrease in central macular thickness was also found in OCT, which raised the speculation of retrograde degeneration. (**D**) ffERG revealed an abnormal response in all three components, which further supported the proposal of retrograde degeneration.

In some complicated cases, ffERGs could provide further diagnostic clues in patients with retinopathy, optic neuropathy, systemic disease, and non-specific visual disturbances. (Figure 5, Figure 6 and Figure 7). In the ‘optic neuropathies’ group, ffERGs were performed to exclude retinal dysfunction, especially for the patients whose symptoms or visual function was worse than the extent of the defect found by imaging studies. A normal ffERG confirms the diagnosis of optic neuropathy confined to retrobulbar structures, while a subnormal ffERG may indicate retinal dysfunction subsequent to severe or long-standing optic nerve damage and an even worse visual prognosis [26]. Weiner et al. found that 37.8% of their glaucoma patients had subnormal ERGs [27]. Moreover, in patients suspected to have glaucoma, photopic negative response in ffERGs serves as an accessory indicator for detecting glaucomatous defects and evaluating the prognosis besides the ordinarily used visual field test and OCT. Retinal function seems compromised under long-standing elevation in intraocular pressure [28,29]. Similarly, central nervous system disease accounted for most of the patients in the “systemic diseases” group, and normal ffERGs confirmed a retrobulbar origin in those suffering from significant visual deterioration, while abnormal ffERGs indicated a poor prognosis [30]. In agreement with our study, ffERGs were most frequently arranged for children with systemic diseases, such as those with epilepsy, mitochondrial diseases, or specific syndromic diseases [24,31]. Considering that the pathogeneses in these patients might involve concurrent systemic and ocular developmental deficits, ffERG enhances the diagnosis and provides useful information regarding retinal development. ffERG is considered to act as a guide for directing differential diagnoses in patients with non-specific or specific visual disturbances, amblyopia, or suspected malinger tendencies.

In addition to the ‘retinal dystrophies’ and ‘other retinal or macular diseases’ groups, which harbored direct retinal pathology, a high abnormal rate (27.3%) was found in the ‘systemic diseases’ group. In this group, abnormal ffERG was most frequently seen in patients under the age of 20 (71.4%, 10/14), and most of them had developmental delay and syndromic diseases. Camuglia et al. also found ERG valuable when evaluating pediatric patients and 34% of them had abnormal results [17]. In contrast, the diagnoses in elderly populations were mostly central nervous system and autoimmune diseases. That is, even in the absence of a particular ophthalmological diagnosis, retinal function can potentially be obliterated simultaneously in systemic diseases. For example, subnormal ffERG has been reported in patients with cerebral autosomal dominant arteriopathy with subcortical infarcts and leukoencephalopathy (CADASIL), which is a severe cerebral vasculopathy [32]. The pathophysiology of cerebrovascular diseases and the retrograde involvement of the retina require more evidence to be established in future research [30]. In the ‘optic neuropathies’ group, the majority of patients under 20 years old with abnormal ffERGs were diagnosed with optic nerve atrophy. The development of optic nerve atrophy may also indicate developmental disorders in the retina and compromised retinal function, but the relationship remains unclear [33]. The highest proportion of patients in the ‘visual complaints’ group had a normal ffERG response and complained of visual disturbance but lacked characteristic image findings. A normal ffERG result generally confirms proper retinal function. In contrast, despite being relatively abnormal, ffERG reports were still inconclusive in one-tenth of the patients in this group. Further investigations and follow-ups are required to explain the observed ffERG responses.

There were some limitations in our study. Because of its retrospective design, the follow-up periods varied between patients. We attempted to make precise clinical diagnoses based on our data; however, we still believe that the database is valuable because, to the best of our knowledge, this is the largest series to appraise the role of ffERG during the past two decades, when diagnostic images have been widely applied in clinical settings. This study illustrates the application of ffERG in the ophthalmologic department of a tertiary referral center, and it remains to be widely used in modern ophthalmological clinics. We suggest that ffERG is essential for clinical diagnosis because it is the test of choice for evaluating retinal function and guiding further surveys for a wide variety of ocular and systemic diseases.

## Figures and Tables

**Table 1:** Patient diagnoses and their age distributions at their first full-field ERG in the six diagnosis groups.

	Total	Age (Years)								
		<10	10–20	20–30	30–40	40–50	50–60	60–70	70–80	>80
Diagnosis Group	n (% of Group)	n	n	n	n	n	n	n	n	n
Retinal dystrophies										
Retinitis pigmentosa	367 (60.8)	5	25	38	69	74	71	60	21	4
Macular dystrophy	85 (14.1)	3	13	11	12	17	12	12	4	1
Cone–rod dystrophy	30 (5.0)	1	4	2	9	4	4	5	1	0
Leber’s congenital amaurosis	29 (4.8)	18	6	1	2	1	0	1	0	0
Bietti crystalline dystrophy	16 (2.6)	0	0	0	4	7	4	1	0	0
PPVRCA	13 (2.2)	0	1	1	1	2	3	4	1	0
Family history	9 (1.5)	0	1	2	2	3	1	0	0	0
Cone dystrophy	8 (1.3)	4	0	1	1	1	1	0	0	0
Occult macular dystrophy	8 (1.3)	0	2	1	2	1	2	0	0	0
Achromatopsia	7 (1.2)	0	2	2	0	1	1	0	1	0
Choroideremia	5 (0.8)	0	0	2	1	0	1	0	1	0
AZOOR	4 (0.7)	0	0	2	1	1	0	0	0	0
Retinoschisis	4 (0.7)	1	2	1	0	0	0	0	0	0
CSNB	3 (0.5)	0	1	2	0	0	0	0	0	0
Albinism	2 (0.3)	0	0	2	0	0	0	0	0	0
Carrier	2 (0.3)	1	0	0	1	0	0	0	0	0
Other dystrophies	12 (2.0)	2	0	2	2	2	0	2	2	0
Other retinal or macular diseases										
AMD	82 (24.6)	0	0	0	2	2	20	28	21	9
Vitreomacular disorders	70 (21.0)	2	5	0	9	10	18	18	6	2
Cancer-related	57 (17.1)	0	0	0	4	14	9	17	9	4
Inflammatory disease	32 (9.6)	0	0	4	13	6	5	4	0	0
Myopic degeneration	30 (9.0)	1	3	2	5	8	5	2	4	0
Post-RD operation	25 (7.5)	0	1	1	2	4	9	7	1	0
Diabetic retinopathy	16 (4.8)	0	0	1	1	1	4	4	5	0
Vascular disease	15 (4.5)	0	0	0	1	1	4	7	2	0
Uncertain retinopathy	7 (2.1)	0	0	0	1	1	3	2	0	0
Optic neuropathies										
Optic neuropathy	133 (100)	4	10	11	12	30	28	14	16	8
Systemic diseases										
Neurological disease	98 (89.1)	5	7	10	11	16	14	19	12	4
Autoimmune disease	3 (2.7)	0	0	1	0	1	0	1	0	0
Metabolic disease	3 (2.7)	0	0	1	1	0	0	1	0	0
Endocrine disease	2 (1.8)	0	1	0	0	0	0	1	0	0
Mitochondrial disease	2 (1.8)	0	1	0	0	1	0	0	0	0
Psuedoxanthoma elasticum	1 (0.9)	0	0	0	0	0	0	0	1	0
Hematology disease	1 (0.9)	0	0	0	0	1	0	0	0	0
Visual complaints										
Non-specific visual disturbances	249 (80.8)	7	16	31	36	51	53	43	10	2
Suspected night blindness	27 (8.8)	0	1	7	6	5	5	2	1	0
Suspected retina origin amblyopia	22 (7.1)	8	4	5	0	0	2	3	0	0
Malinger tendency	10 (3.2)	1	2	4	1	0	1	1	0	0
Others										
Clinical study	79 (47.6)	0	0	1	3	10	17	31	14	3
Drug-associated	67 (40.4)	0	3	9	6	12	15	13	7	2
Trauma	14 (8.4)	0	1	1	2	2	0	7	0	1
Pre-cornea transplantation evaluation	6 (3.6)	0	0	0	0	2	1	1	1	1
Total ERG	1655	63	112	159	223	292	313	311	141	41

ERG, electroretinography; PPVRCA, pigmented paravenous retinochoroidal atrophy; AZOOR, acute zonal occult outer retinopathy; CSNB, congenital stationary night blindness; AMD, age-related macular degeneration; Post-RD, post-retinal detachment.

## Data Availability

The data presented in this study are available on request from the corresponding author. The data are not publicly available due to privacy.
